# The contribution of childhood adversity to cortisol measures of early life stress amongst infants in rural India: Findings from the early life stress sub-study of the SPRING cluster randomised controlled trial (SPRING-ELS)

**DOI:** 10.1016/j.psyneuen.2019.05.012

**Published:** 2019-09

**Authors:** Sunil Bhopal, Deepali Verma, Reetabrata Roy, Seyi Soremekun, Divya Kumar, Matt Bristow, Aparna Bhanushali, Gauri Divan, Betty Kirkwood

**Affiliations:** a Maternal & Child Health Intervention Research Group, Department of Population Health, Faculty of Epidemiology & Population Health, London School of Hygiene & Tropical Medicine, United Kingdom; b Sangath, New Delhi, India; c School of Psychology and Sports Science, Anglia Ruskin University, Cambridge, United Kingdom; d Research & Development Division, SRL Laboratories Ltd, Mumbai, India

**Keywords:** Infant, Child, Adversity, Adverse childhood experiences, Stress, HPA axis, Cortisol

## Abstract

**Background:**

The majority of the world’s children live in low- and middle-income countries and face multiple obstacles to optimal wellbeing. The mechanisms by which adversities – social, cultural, psychological, environmental, economic – get ‘under the skin’ in the early days of life and become biologically embedded remain an important line of enquiry. We therefore examined the contribution of childhood adversity through pregnancy and the first year of life to hair and salivary cortisol measures of early life stress in the India SPRING home visits cluster RCT which aims to improve early childhood development.

**Methods:**

We assessed 22 adversities across four domains: socioeconomic, maternal stress, family-child relationship, and child and summed them to make a cumulative adversity score & quintiles, and four subscale scores. We cut 3 cm of hair from the posterior vertex and took three saliva samples from morning till late afternoon on each of two days (total six samples). We analysed both for cortisol concentration using ELISA techniques. We used multiple linear regression techniques to assess the relationship between cumulative adversity and log hair cortisol concentration and saliva diurnal slope and area under the curve.

**Results:**

We assessed 712 children for hair, and 752 children for saliva cortisol at 12 months of age. We found a strong positive relationship between adversity and hair cortisol; each additional adversity factor was associated with hair cortisol increases of 6.1% (95% CI 2.8, 9.4, p < 0.001) and the increase from adversity quintile one to five was 59.4%. Socioeconomic, relationship and child scales were independent predictors of hair cortisol (socioeconomic 6.4% (95% CI -0.4, 13.6); relationship 11.8% (95% CI 1.4, 23.2); child 7.9% (95% CI -0.5, 16.9). We did not find any association between any measures of adversity and either of the saliva cortisol outcomes.

**Discussion:**

This is the largest study of hair cortisol in young children, and the first in a low- and middle-income country setting. Whilst the short-term diurnal measures of cortisol did not appear to be linked with adversity, chronic exposure over several months appears to be strongly associated with cumulative adversity. These findings should spur further work to understand the specific ways in which adversity becomes biologically embedded, and how this can be tackled. They also lend support to ongoing action to tackle childhood adversity in communities around the world.

## Highlights

• Hair cortisol concentration was associated with childhood adversity in one year olds in rural India.
• Saliva cortisol slope and area under the curve did not show this association.
• This is the largest study of hair cortisol in young children.
• This is the first study to report hair cortisol from a low/middle income country.

## 1 Introduction

Health and development in the crucial early life period is now firmly on the global agenda and interventions are being designed to address the myriad obstacles to optimal wellbeing faced by the majority of the world’s children who live in low- and middle-income countries. Childhood adversity is associated with negative effects across the lifecourse including on growth, development, behaviour and academic ability in childhood and conditions including depression, cardiovascular disease and healthy ageing later in life (Shonkoff et al., 2012). The question, then, is by which mechanisms do these disparate adversities – social, cultural, psychological, environmental, economic – get ‘under the skin’ and become biologically embedded (Berens et al., 2017; Nelson, 2013)? Several mechanisms have been proposed and studied including genetics & epigenetics, inflammation, hormonal changes, and structural & functional brain changes.

Cortisol is one of the most studied of these mechanisms, and we examined the relationship between this hormone and childhood adversity in young infants aged 12 months in India. Cortisol is the end product of the hypothalamic-pituitary-adrenal (HPA) axis and has been widely used as a stress biomarker, but rarely in such young infants. It can be measured in multiple ways. The concentration of cortisol in hair gives a measure of chronic exposure to stress over a period of months (Russell et al., 2012). Measuring cortisol in multiple saliva samples at different times of day allows for measures of diurnal change to be calculated – cortisol is expected to be highest upon waking and falls through the day (Adam and Kumari, 2009). These measure two complementary features of a healthy cortisol rhythm – that it should fall from a peak soon after waking to a nadir in the evening (slope), and that increased exposure throughout the day will lead to elevated cortisol area under the curve (Area under the curve). This rhythm starts in the first six months of life and is expected to be fully established by age one year (Gunnar and Adam, 2012; Mantagos et al., 1998). Other developmental consideration include daytime naps (Watamura et al., 2004) which must be accounted for in this age group. Few studies examine associations between hair cortisol and cortisol in other samples in the first year of life.

Changes in cortisol are well documented in older children exposed to adversities including those exposed to deprived care in Romanian orphanages (Gunnar et al., 2001) where flattened diurnal rhythm is described in saliva cortisol at age 6–12 years following adoption in the first year of life, maternal depression and participation in home visiting programmes (Fernald and Gunnar, 2009) where participation in a home-visiting programme was associated with lower salivary cortisol at age 2–6 years, and childhood trauma is associated with increased mean cortisol in a range of studies reviewed by Nemeroff (Nemeroff, 2004). A recent systematic review included 36 studies of hair cortisol in children from birth-18 years of age describing higher hair cortisol in boys, and with greater body mass index. A possible association with socioeconomic status was described whereby higher socioeconomic status was associated with lower hair cortisol and the authors called for more research into associations with stressful experiences (Gray et al., 2018). Another recent review examined diurnal salivary cortisol measures in all age groups and described associations of flattened diurnal slopes with impaired health outcomes; this relationship was clearer in adults than in children (Adam et al., 2017). A systematic review focussing on young children aged 12–60 months included nine studies and found chronic stress was associated with raised hair cortisol but that there was no difference between boys and girls at this age (Bates et al., 2017).

In the work described in this paper we therefore set out to assess the relationship between early life adversity and these chronic & diurnal measures of cortisol in hair & saliva in children enrolled in the Early Life Stress sub-study of the SPRING cluster randomised controlled trial in rural Haryana, India. Our hypothesis was that adversity would be associated with increased hair cortisol, and in saliva with increased daily exposure to cortisol and flattened diurnal slope. This is the first time hair cortisol has been reported in children from a low/middle-income country (LMIC), and few studies examine both saliva & hair simultaneously.

## 2 Methods

### 2.1 Overview of study design

SPRING-ELS was a sub-study of the Wellcome Trust funded SPRING cluster randomised controlled trial in India. It focussed on cortisol measures of early life stress, and on early childhood adversity in children enrolled in SPRING. Details on SPRING are available elsewhere (Clinicaltrials.gov registration NCT02059863) but in brief SPRING developed an innovative, feasible, affordable & sustainable community-based approach to delivering a home visiting programme aiming to improve child growth & development at-scale in India & Pakistan, two countries with high burden of disadvantage. Implementation was evaluated by parallel cluster randomised controlled trials where clusters represent geographical areas served by a health sub-centre with a functioning auxillary nurse midwife, covering a population of at least 8000. The primary outcomes were height-for-age, the best early childhood predictor of human capital (Victora et al., 2008), and Bayley Scales of Infant Development III, the gold standard assessment of a child’s development in the early years (ClinicalTrials.gov: SPRING Cluster Randomised Controlled Trial, 2018). These impact outcomes were complemented by in-depth economic analysis, process-evaluation and a broad range of intermediate outcomes selected based on a predefined conceptual-framework. The aim was to help to unpack the SPRING causal pathway, provide deeper understanding of mechanisms of trial impact, and inform lessons for scale-up and incorporation into health systems. SPRING took place in 120 villages of Rewari district, Haryana state across a population of around 200,000. The district is predominantly rural and has health and demographic indicators around average for Haryana state. The literacy rate in Haryana is 76%, with female literacy of 67%. The sex ratio is 879 females per 1000 males – amongst the lowest ratio in India (Office of the Registrar General & Census Commissioner, India Ministry of Home Affairs, Government of India, 2015). Infant mortality is 41/1000 live births (National Institution for Transforming India, Government of India, 2016) – around the national average. More than one third of under-five year old children are stunted (National Family Health Survey (2017)).

### 2.2 Data collection

Children were identified by a surveillance system whereby trained resident fieldworkers visited each household in the study area every 8 weeks to identify pregnancies and births, and follow-up pregnant women & children already identified. Socioeconomic data was collected at enrolment and assessors were trained to take saliva and hair samples from children, and to do adversity assessments with their mothers when infants turned 12 months of age. Full implementation of the SPRING intervention was achieved in May 2015 and these children were born from 18 June 2015. Assessments were therefore done when SPRING had been running for at least one year in intervention clusters.

#### 2.2.1 Adversities

These were selected to be contextually important based on formative research with local mothers and grandmothers, advice from child development experts and reviews of the literature on existing tools. The adversities covered four domains as follows: 1) household-level socio-economic factors, 2) maternal stressors, 3) child-carer relationships and 4) child-related factors. The focus was on these groups because young children in this setting spend most of their time and interact most closely with family members inside the home. The aim was to focus on a broad range of potential impediments to wellbeing because children can be resilient to single adversities, but combinations of these may be more harmful and overwhelm protective factors in a child’s life (Wachs and Rahman, 2013).

Data on 22 adversities were chosen. These are summarised in Table 1 which shows the four domains in which they were placed, and the prevalence of each adversity. Nineteen of the adversities were assessed at one year of age and only the first three (marked *E in the table) were assessed at enrolment. Further details on each adversity are provided below. These descriptions were published previously (Bhopal et al., 2019).

**Table 1:** 22 Childhood adversities within four categories: socioeconomic, maternal stress, relationship & stress.

Domain	Item	Prevalence^h^
Socioeconomic	Socioeconomic status: lowest quintile (*E) a Father education: none or 1-5 grades (*E) Mother education: none or 1-5 grades (*E) Father occupation: at home, seasonably employed or casual labourer Mother married under legal age (18 years) Family debt b or mother reports being unable to afford food for self or child at any point c	20.0% 5.0% 11.9% 24.7% 20.0% 18.0%
Maternal Stress	Mother reports death of husband, parent, sibling, child or friend since pregnancy Mother seriously injured or ill since pregnancy Any violence from husband or mistreated by any other person since pregnancy d PHQ9 score > = 5 or problems described make it very/extremely difficult to do daily activities Duke scale: support < = 40 or stress >27 Husband’s alcohol use causes problems for mother e	5.4% 4.0% 13.4% 19.5% 6.3% 8.3%
Relationship	Any of mother, father, mother or mother-in-law were “unhappy” when found out child was a girl f MORS concern: moderate or high Observed feeding style: very low quality HOME score: lowest quintile	15.2% 50.4% 13.3% 15.6%^g^
Child	Mother-reported child born early Child admitted to hospital any time after birth Mother & child separated for one week or more Child left alone or with child under 10 years for more than one hour in the past week Older children who live in house: say anything to make child cry or unhappy (in last week) Older children who live in house hit/punched/kicked/bit child on purpose to make them unhappy (in last week)	10.2% 14.9% 1.7% 4.6% 30.5% 17.9%

a SES score calculated with principle components analysis using data on mother & household demographics and animal & asset ownership.

b Answered yes to question: “Since you became pregnant, have you or your immediate family who live with you been in debt?”.

c Answered yes to question: “Since you became pregnant, have you ever been hungry because you could not afford to buy food?” or similar related to child.

d Using WHO multi-country study on women’s health and domestic violence against women(World Health Organization, 2005).

e If woman reported husband drinking alcohol, answered yes to question: “does this cause any problems for you”.

f Question: “When [person] found out your baby was a girl were you/they happy, unhappy or didn’t mind whether you had a girl or a boy?”.

g Not exactly 20% because cut-off made at change between integers (HOME score of 27 & 28).

h Includes all children with adversity assessments regardless of hair or saliva assessment status. No imputation for missing values.

* E data collected at enrolment; all others collected at 12 m.

**Socioeconomic:**

1) Asset index - being in the lowest quintile for the population at enrolment (calculated with principal components analysis using data on mother, household demographics and animal & other asset ownership) 2) Low parental education – no education or primary-schooling only (asked at enrolment) 3) Father occupation - father did not work, was seasonably employed or was a casual labourer at 12-month assessment 4) Mother married under the legal age of 18 years 5) Family debt - mother reported family debt or being unable to afford to buy food for herself or her child at any point between becoming pregnant and the 12 month assessment.

**Maternal stress:**

1) Death of one or more of mother’s close family members since becoming pregnant 2) Mother seriously injured or ill since pregnancy 3) Any violence towards mother from husband (assessed using WHO multi-country study on women’s health and domestic violence against women (World Health Organization, 2005)) or any other person since becoming pregnant 4) mother screens positive for mild, moderate or severe depression on PHQ9 or answers ‘yes’ to PHQ9 question on suicidal ideation (at 12-month assessment). PHQ9 is one of the most commonly used screening tools for depression and has been used widely in India (Patel et al., 2010) 5) Low level of support or high stress from others around the mother using the Duke social support & stress scale (Parkerson et al., 1991) reported at 12-month assessment 6) Problematic husband alcohol use reported by mother at 12-month assessment

**Relationship:**

1) Any family member was unhappy when they found out that the child was a girl 2) Moderate or high concern level on Mother Object Relations Scale – short form (MORS-SF) at 12-month assessment. MORS-SF is a screening tool consisting of 14 short statements which a mother is asked to rate on a Likert-type scale to identify potential problems in early mother-infant relationship (Oates et al., 2005) 3) Very low quality interactions observed during a feeding episode at the 12-month assessment (assessed by non-specialist fieldworkers using the observed feeding index, a tool developed in this project where feeding is scored using tick-boxes. Assessor-expert reliability tests done using videos showed an overall reliability of 90% for all items with more than 80% agreement for each assessor. This tool will be published in due course). Very low quality means that the following was observed during the feeding episode: < = 1 positive talk by mother towards child, and < = 1 episodes of playful feeding and < = 1 responsive feeding actions, plus one or more negative actions such as force feeding, holds child’s head still to give food, shaking, threatening, shouting or berating observed by the mother towards child during feeding session 4) Lowest quintile score on HOME inventory measuring quality of the home environment through observations of the home and questions to the mother (total of 45 items, each scored 0 or 1) over the course of one hour (Cox and Walker, 2002) at 12 month assessment – the cut-off for the quintile fell between 27 & 28 points and the lowest of these (27 points) was chosen to create a conservative estimate of this factor.

**Child:**

1) Child born prematurely (asked at 12-month assessment) 2) Child hospitalised in first year of life 3) Separation of mother & child for more than a week in the first year of life 4) Inadequate care – child left alone or with a child under 10 years for more than one hour in the past week (assessed at 12-month assessment) (From (“UNICEF: Multiple Indicator Cluster Surveys,” 2018)) 5) Older children in the house say anything to make child cry or unhappy (in last week) (at 12-month assessment) (From (“UNICEF: Multiple Indicator Cluster Surveys,” 2018)) 6) Older children who live in house: hit/punched/kicked/bit child on purpose to make them unhappy (in last week) (assessed at 12 month assessment)

Adversity questionnaires were double-entered and verified using a computer program written in C Sharp with an SQL Server 2008 database.

#### 2.2.2 Hair sampling

Trained assessors cut hair samples from the posterior vertex (the area of least intra-individual variability (Sauvé et al., 2007)) as close to the scalp as possible using scissors. The aim was to obtain at least 10 mg of hair (approximately 1 cm diameter, 2–3 cm length) (Stalder and Kirschbaum, 2012). This amount of hair was acceptable to families and caused minimal impact to hair appearance. Samples were wrapped in aluminium foil, labelled and the scalp end marked. On arrival at the site office samples were cut to select the most proximal 3 cm of hair and repackaged in aluminium foil then a paper envelope ready for weekly courier collection and shipping to the laboratory at room temperature. On arrival at the laboratory, samples were stored at room temperature and analysed using established methods (Davenport et al., 2006; Kirschbaum et al., 2009) whereby the hair is washed in isopropanol, dried thoroughly for 24 h, cut finely with scissors, extracted into methanol and analysed using a Salimetrics ELISA kit to give a final result for each hair sample in picograms of cortisol per milligram of hair. The laboratory protocol is presented as an additional file.

#### 2.2.3 Saliva sampling

The same fieldworkers took saliva samples three times (at 8am, 12 pm and 4 pm) on each of two consecutive days (total six samples). They used Salimetrics SalivaBio Children’s Swabs (Salimetrics, USA; part no 5001.06) which are designed for young infants to avoid choking risk and to be palatable. These swabs have been used in many settings worldwide and are the gold-standard for collection of saliva for cortisol analysis (Tryphonopoulos et al., 2014). The first sample of each day was taken as soon as possible on entering the household in order to minimise the opportunity for there to be a transient rise in concentration due to a stranger in the household. Samples were not taken if children had been unwell in the past 24 h because illness can lead to raised cortisol. Samples were never taken within 30 min of eating drinking or waking from sleep to avoid interference with cortisol levels (Schwartz et al., 1998).

Sampling was done as follows. The child was positioned on their mother or grandmother’s lap and the fieldworker gently introduced the swab into the child’s mouth for 30–60 s. When it was observed to be at least 1/3 saturated (a minimum of 150 μL saliva is required for laboratory analysis) it was removed, placed into a storage tube (Salimetrics USA; part no 5001.05) and labelled with a pre-printed freezer-proof label containing only a sample identifier and anonymised child identifier. Time of sampling, time of last food, drink & last waking were then recorded.

Samples were kept cool in insulated flasks through the day and refrigerated them at the site office overnight (samples remain stable at room temperature for several weeks (Nalla et al., 2015; Tryphonopoulos et al., 2014) but cooling is normal practice) before being packed in a cooled container (2–5 °C) for daily courier collection and shipping to SRL Laboratories Ltd, Mumbai, India. Shipping followed the laboratory’s established freight route by road to Delhi airport (1.5–2 h), and air to Mumbai (approximately 2 -h flight) from where they were delivered to the laboratory’s Research & Development division. Samples were frozen on arrival and stored at −20 °C. Samples were thawed in batches, centrifuged at 1500 *g* for 15 min and refrozen at −20 °C. Samples were analysed later in batches using a Salimetrics USA high-sensitivity salivary cortisol enzyme-linked immunosorbent assay (ELISA) according to the manufacturer’s instructions. A randomly selected 10% of samples per batch were analysed in duplicate. The intra-assay coefficient of variation of 5.6% and inter-assay coefficient of variation of 9.2% was within acceptable limits (Salimetrics, 2018).

### 2.3 Sample size

With 24 geographical clusters, a sample size of 25 children per cluster was chosen to give 90% power at the 5% level of significance to explore a range of adversities with prevalence of 20%–80% and to detect effect sizes between 0.4SD & 0.5SD (assuming an intra-cluster correlation of 0.05). The aim was to assess more children than this - at least 30 children per cluster for saliva and 35 per cluster for hair - to ensure that the minimum sample size was met even if samples were of insufficient volume (saliva) or weight (hair) for analysis.

### 2.4 Data analysis

Stata 15 was used for all analyses (StataCorp LLC: College Station, TX, USA).

#### 2.4.1 Adversities

we used multiple imputation by chained equations (MICE) to account for the missing values in adversity data described in Table 2. We used 30 imputations and included all explanatory and outcome variables in each model as is standard practice. We also carried out a repeat analysis using only complete cases. We calculated descriptive data using a combination of all imputations.

**Table 2:** Adversity scores - proportion with missing values in each of hair & saliva sub-samples.

Adversity	Number (%) children in each sub-sample with missing values	
	Hair	Saliva
Mother marriage age	16 (2.2%)	14 (1.9%)
PHQ9 score	13 (1.8%)	18 (2.4%)
Duke scale	13 (1.8%)	18 (2.4%)
Observed feeding index	201 (28.2%)	176 (23.4%)
HOME-IT score	0 (0%)	1 (0.1%)

We categorised adversities in three ways as follows: 1) we summed the adversities to create a total adversity score of 0–22 following a cumulative-adversity model (Björkenstam et al., 2017; Slopen et al., 2014; Turner and Lloyd, 1995) 2) we summed adversities within each of the four domains in a similar manner 3) we used principle-components-analysis (PCA) to capture the linear combination of adversities which creates the maximum variance in the adversity data to avoid any ‘double counting’ in the cumulative adversity analysis. We converted the raw PCA score into adversity quintiles for analysis.

#### 2.4.2 Hair cortisol

We log-transformed hair cortisol concentrations for each child because of left-skew and then winsorized four remaining outliers to 3SD above the mean. This was the first outcome variable.

#### 2.4.3 Saliva cortisol

We calculated two outcomes from saliva cortisol measures for each child. The first was saliva cortisol slope which is a measure of the change in saliva cortisol concentration per hour across the day’s sampling. The second was saliva cortisol area under the curve which is a measure of the total hourly exposure of a child to cortisol over the sampling period. For each saliva cortisol result we first winsorized outlying high values to 3SD above the mean. We then used the rise-over-run formula (change in two cortisol values divided by hours between these) for children with results at 8am and 4 pm to calculate the saliva slope for each child. Similarly, we used the trapezoid formula to estimate the total cortisol a child was exposed to which was represented by the total area bounded by two parallel lines at each of two time points on the x-axis, the base on the y axis (where cortisol is zero) and the line connecting the two cortisol values on the y-axis. This was done for children with all three samples on at least one day to calculate saliva cortisol area under the curve with respect to the ground.

### 2.5 Association of cumulative adversity and hair cortisol

We assessed the relationship between cumulative adversity and hair cortisol using multi-level modelling, accounting for clustering as a random-effect and allocation to the SPRING intervention or control arm allocation as a binary fixed-effect in the model. We first treated adversity as categorical to examine mean hair cortisol for each of the observed cumulative adversity scores, and then as continuous to assess the linear trend in this relationship. We ran the same model with the adversity quintiles replacing cumulative adversity. We ran all of these models including Sex and Body Mass Index (BMI) to assess for possible confounding.

### 2.6 Association of cumulative adversity and saliva cortisol outcomes

We also used multi-level modelling to assess the association of adversity and saliva cortisol slope in the whole sample using a three-level model which accounted for saliva results nested hierarchically within the random effects day, child & cluster, accounting for time of sample collection as a fixed-effect interaction term. Child wake-up time was a fixed-effect to control for any effect of waking time on saliva cortisol values. The difference in saliva cortisol slope between cumulative adversity scores was represented by the interaction term between adversity and time of sample with cortisol result at that time as the outcome.

We used a similar approach to model AUC. The difference in AUC between cumulative adversity scores for the whole sample was modelled using predicted margins for cortisol concentration at 8am, noon & 4 pm. The model first used the trapezoid formula described earlier to calculate the AUC at each adversity level, and then subtracted one from the other to calculate the difference. We ran these models including Sex and Body Mass Index (BMI) to assess for possible confounding.

### 2.7 Association of adversity domains and cortisol

We examined the association between the four adversity domains and the three cortisol outcomes using similar models to the analyses with total cumulative adversity. We used a three-step process as follows:

We first ran models to explore the association between each domain and cortisol, adjusted only for clustering and trial arm allocation. We then added socioeconomic score to the models for Maternal Stress, Relationship & Child domains to adjust for potential confounding in these associations by socioeconomic status. We finally ran a model including all scales showing either a strong relationship or p-value less than 0.1 to create a mutually adjusted model.

### 2.8 Ethics

Ethics approval was obtained from the London School of Hygiene & Tropical Medicine research ethics committee (23 June 2011, approval number 5983; 19 May 2015, approval number 9886) and the Sangath Institutional Review board (IRB) (19 February 2014; 27 May 2015). Approval was also granted by the Indian Council of Medical Research’s Health Ministry Screening Committee (HMSC) (24 November 2014; 6 October 2015). The SPRING trial is registered with clinicaltrials.gov, number NCT02059863. Informed written consent was obtained from mothers at identification by the surveillance system and again before a child’s first birthday.

### 2.9 Role of funding source

The funder (Wellcome Trust) had no role in the design and conduct of the study; collection, management, analysis, and interpretation of the data; and preparation, review, or approval of the manuscript. SB & BK have complete access to the study data and are responsible for the reported study findings, and made the decision to submit for publication.

## 3 Results

### 3.1 Hair and saliva cortisol sub-samples

**Fig. 1:**
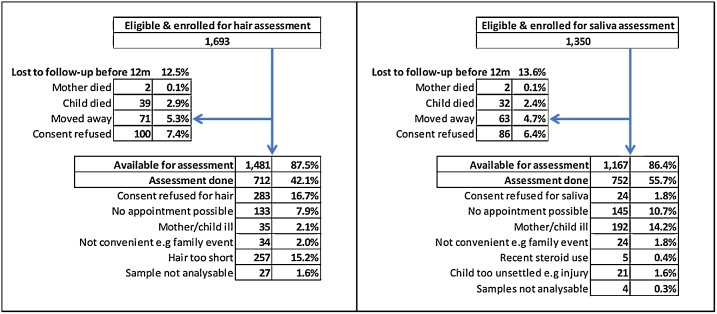
Hair & Saliva Subsample flowchart.

1693 children were enrolled for hair assessment, and 1350 children were similarly enrolled for saliva assessment. The flowcharts for both of these sub-samples are shown in Fig. 1showing that 712 children had hair assessments and 752 had saliva assessments. Loss to follow-up was because of consent refusal, having moved away, being unable to make an appointment and (for hair only) because the hair length was too short for sampling. 436 children had both hair and saliva assessments. The median age at assessment was 12.4 months (IQR 12.2–12.6). All of these children had adversity assessments. Prevalence of each adversity factor ranged from 4.0% to 50.4% (Table 1). The extracted PCA factor explained 10.7% of variance; factor loadings for adversities are presented in Supplementary File 1 alongside a Scree plot.

Table 3 shows that there was no evidence of selection bias with regards to maternal education, caste, socioeconomic scores, being a twin/triplet, and mother’s age at delivery. However, girls were more likely to be assessed than boys in the hair sub-sample. Table 4 shows that correlation between the four adversity domains was greatest for socioeconomic and maternal stress domains, and less for others including socioeconomic and child domains.

**Table 3:** Comparison of children completing hair assessments with those lost to follow-up, and comparison of children with 0, 1 and 2 days of saliva sampling (*adjusted for clustering).

Indicator	HAIR				SALIVA			
	Completed Assessment (C)	Lost to Follow up (L)	C-L Difference (95% CI)*	p*	0 days	1 day	2 days	p*
Numbers meeting inclusion criteria in hair & saliva subsamples	**712**	**981**			**598**	**125**	**627**	
**% No education (n)**	5.3% (38)	7.2% (71)	−1.8% (-4.0, 0.5)	0.147	7% (42)	6.2% (39)	4.8% (6)	0.594
**% scheduled/backward caste/tribe (n)**	59.1% (421)	60.4% (593)	−3.0% (-8.3, 2.3)	0.504	58.7% (351)	61.2% (384)	60% (75)	0.750
**% poorest (lowest 2 quintiles) (n)**	39.7% (283)	42.7% (419)	−2.5% (-7.5, 2.4)	0.179	42.1% (252)	44.5% (279)	47.2% (59)	0.560
**% Male (n)**	47.9% (341)	58.4% (573)	−10.5% (-15.4, -5.7)	<0.001	55% (329)	53.7% (337)	49.6% (62)	0.541
**% Twins/Triplets (n)**	1.3% (9)	1.6% (16)	−0.2% (-1.0, 0.6)	0.219	0.8% (5)	2.4% (15)	0.8% (1)	0.093
**% Delivered in facility (n)**	97.9% (697)	97.5% (956)	0.4% (-1.0, 1.9)	0.852	97.5% (583)	97.6% (612)	97.6% (122)	0.991
**Mean age at delivery (sd)**	22.3 (3.7)	22.3 (3.8)	0.02 (-0.34, 0.38)	0.842	22.2 (3.5)	22.5 (3.9)	22.2 (3.7)	0.362
**Mean SES score (sd)**	0.05 (2.6)	−0.4 (2.9)	−0.02 (-0.28, 0.25)	0.931	−0.14 (2.93)	−0.36 (2.56)	−0.24 (2.77)	0.550

**Table 4:** Correlation between adversity domains.

DOMAIN	Socioeconomic	Maternal Stress	Relationship	Child
Socioeconomic	1.00	–	–	–
Maternal Stress	0.47	1.00		
Relationship	0.34	0.24	1.00	
Child	0.06^a^	0.15	0.14	1.00

a All correlations p < 0.001 except that marked ^a^ which is p = 0.172.

**Fig. 2:**
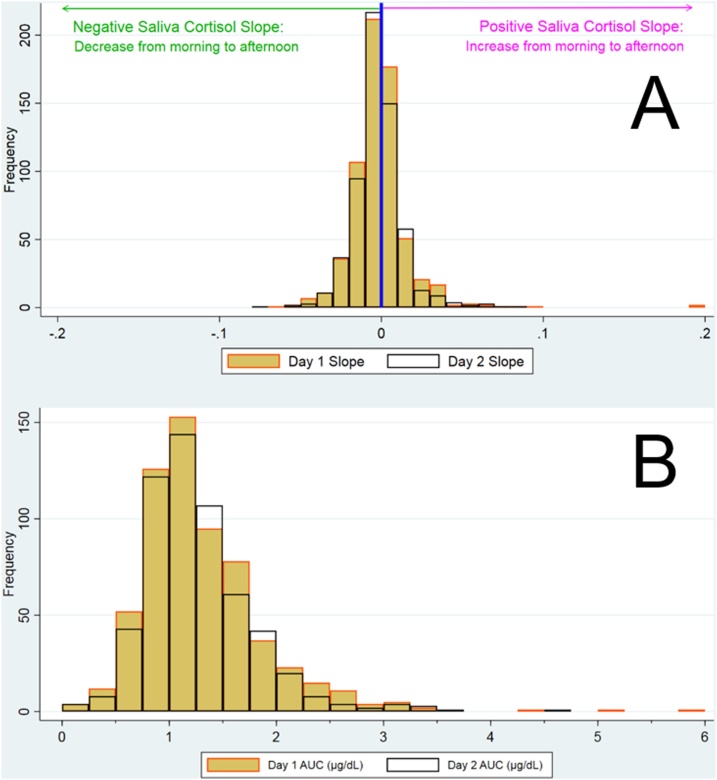
Saliva cortisol descriptive analysis: slopes (A) & area-under curve (B) for days 1 and 2.

Table 5 shows the mean values for all cortisol measures including for boys and girls separately. The mean of log hair cortisol concentration was 1.85 log pg/mg (SD 1.05) This is equivalent to a geometric mean of 6.26 pg/mg (SD 3.01). Hair cortisol did not vary by length of hair sampled or weight of hair used in analysis. Saliva cortisol slopes were relatively flat; 60.0% of children had a slope between -0.01 μg/dL/hr and +0.01 μg/dL/hr as illustrated in Fig. 2 A. In addition, contrary to our expectation of negative slopes, 15.3% had slopes that increased by more than 0.01 μg/dL/hr through the day. The overall mean saliva AUC was 1.29 ug/dL (SD 0.47). The distribution for saliva cortisol is illustrated in Fig. 2, and this figure also shows little difference in saliva cortisol between the two days of sampling.

**Table 5:** Hair cortisol concentration, saliva cortisol slope & AUC – descriptive data.

	BOYS		GIRLS		OVERALL	
	N	Mean (SD)	n	Mean (SD)	n	Mean (SD)
Hair cortisol concentration* (log pg/mg hair)	341	6.26 (3.01)	371	6.29 (2.71)	712	6.28 (2.85)
**Saliva Slope (ug/dL/hr)**	399	0.00015 (0.022)	353	−0.0023 (0.016)	752	-.0010 (0.019)
**Saliva area under curve** **(ug/dL)**	399	1.29 (0.50)	353	1.28 (0.42)	752	1.29 (0.47)

* Geometric mean.

### 3.2 Hair Cortisol & Adversity

#### 3.2.1 Cumulative adversity

**Fig. 3:**
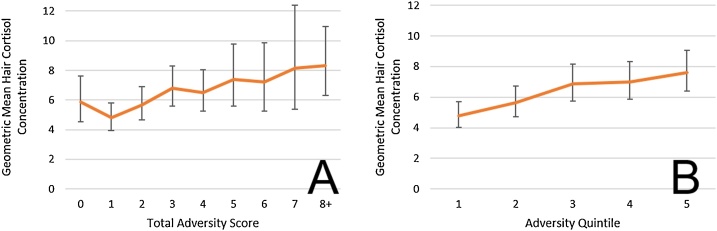
Association of log Hair Cortisol Concentration and Adversity using total adversity score (A) and adversity quintile (B).

Most children had a cumulative adversity score of at least one. The maximum score was 12. Cumulative adversity was strongly positively associated with hair cortisol on the log scale as illustrated in Fig. 3A. The adversity quintile analysis displayed in Fig. 3B shows a similar association; the increase between the least and most adverse quintiles was 59.5% (4.77–7.61 log pg/mg hair). Each additional adversity was associated with an increase of 6.1% (95% CI 2.8, 9.4, p < 0.001) in hair cortisol (bottom shaded row of Table 6). BMI and Sex were not independently associated with hair cortisol, and adding these to the model did not materially change the associations seen.

**Table 6:** Adversity domains and cumulative adversity – association with log hair cortisol concentration.

HAIR CORTISOL CONCENTRATION	n	No of factors	Range observed	Model adjusted for clustering			Model adding socioeconomic			Mutually adjusted model			
				% cortisol increase per adversity	95% CI	p	% cortisol increase per adversity	95% CI	p	% cortisol increase per adversity	95% CI	p	p for model
**Socio-economic factors**	712	6	0-6	8.8%	(2.0, 16.0)	0.011	–	–	–	6.4%	(-0.4, 13.6)	0.065	<0.001
**Maternal stress factors**	712	6	0-4	6.0%	(-3.2, 16.0)	0.207	–	–	–	–	–	–	
**Relationship factors**	712	4	0-3	15.8%	(5.4, 27.2)	0.002	13.3%	(2.8, 24.8)	0.011	11.8%	(1.4, 23.2)	0.026	
**Child factors**	712	6	0-5	10.0%	(1.6, 19.2)	0.019	9.4%	(1.0, 18.5)	0.027	7.9%	(-0.5, 16.9)	0.065	
**Cumulative adversity**	712	22	0-12	6.1%	(2.8, 9.4)	<0.001							

#### 3.2.2 Adversity domains

**Fig. 4:**
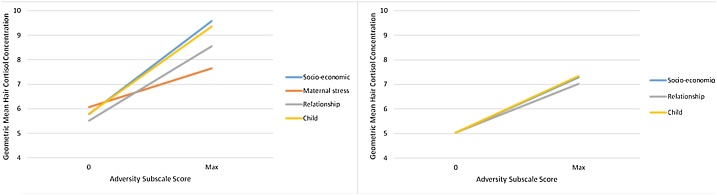
Relationship between adversity subscales and mean hair cortisol concentration – presenting unadjusted (A) and fully-adjusted (B) models.

The strongest association was with the Relationship scale where each increase in score was associated with an increase of hair cortisol of 15.8% on the log scale. This scale was observed across a range of 0–3 adversities and so is not directly comparable to other scales which were observed over greater ranges as illustrated in Table 6. We therefore present Fig. 4 where each scale has been rescaled to between 0 and maximum, allowing for direct comparison. Here, socioeconomic, child and relationship scales have the greatest change in cortisol between those with the least and most adversity, whilst that for maternal stress is lower.

Table 6 also shows that including socioeconomic status in the model ameliorated the relationship between the relationship & child scales and hair cortisol somewhat. The final mutually adjusted model which aims to assess the relative contribution of each domain is presented in this table and suggests that socioeconomic, relationship and child scales were independent predictors of hair cortisol despite 95% CI that cross 0 – the increase in hair cortisol per adversity factor was 6.4% (95% CI -0.4, 13.6, p = 0.065) for socioeconomic, 11.8% (95% CI 1.4, 23.2, p = 0.026) for relationship, and 7.9% (95% CI -0.5, 16.9, p = 0.065) for Child. The p for the model was <0.001. The initial and final models for each adversity domain and hair cortisol are illustrated in Fig. 4.

### 3.3 Saliva Cortisol & Adversity

Analyses presented in Table 7 show that whilst the direction of the effect size for the relationship between saliva cortisol slope & AUC and measures of adversity were all in the hypothesised direction (less steep slopes, and higher AUC), the effect sizes were small with wide confidence intervals meaning that no association was seen between saliva cortisol and measures of adversity.

**Table 7:** Adversity domains and cumulative adversity – association with saliva cortisol slope and area-under-curve.

	n	Number of factors	Range observed	Saliva Cortisol Slope			Saliva Cortisol Area-Under-Curve		
				Increase per adversity	95% CI	p	Increase per adversity	95% CI	p
**Socio-economic factors**	752	6	0-6	0.00009	(-0.00063, 0.00081)	0.805	−0.028	(-0.102, 0.046)	0.456
**Maternal stress factors**	752	6	0-4	0.00021	(-0.00082, 0.00125)	0.683	−0.026	(-0.132, 0.079)	0.626
**Relationship factors**	752	4	0-3	0.00010	(-0.00097, 0.00118)	0.852	−0.030	(-0.140, 0.080)	0.594
**Child factors**	752	6	0-5	0.00063	(-0.00025, 0.00151)	0.162	−0.018	(-0.108, 0.073)	0.703
**Total adversities**	752	22	0-12	0.00017	(-0.00019, 0.00054)	0.352	−0.018	(-0.123, 0.090)	0.362

## 4 Discussion

We found that childhood adversity was clearly positively associated with the concentration of cortisol in hair samples taken at one year of age. This assessed cortisol exposure over several months. This relationship was not confounded by BMI or sex. We did not find the same relationship with saliva measures which focus on the cortisol rhythm over two days. This increased chronic exposure to cortisol is likely to be detrimental to child wellbeing and our findings should serve as a wake-up call that children are never “too-young” to be affected by adverse circumstances, that children need protection & support to avoid adversity and that programmers & policy-makers should reiterate efforts to ameliorate these effects.

Most studies of hair cortisol in young children are relatively small. We identified 11 studies done in under 5 year olds, a similar number to those identified in a recent systematic review (Gray et al., 2018). Most of these had a sample size of less than 100 and none were done in low/middle income countries. Our study is therefore to the best of our knowledge not only one of the largest studies of hair cortisol in young children ever done, but the first in the low/middle-income country setting where the burden of adversity is greatest and where most children live. This limited literature of 0–5 year olds has mixed findings. In concordance with our findings, no association is seen between hair cortisol by gender in these 0–5 year olds ((Gerber et al., 2017; Grunau et al., 2013; Maurer et al., 2016; Rippe et al., 2016) although this has been reported in older children). Two small studies reported no association with socioeconomic status, parental education or income (Groeneveld et al., 2013; Hoffman et al., 2017) whilst a larger study from Canada reported negative associations with parental education and no association with parental income (Vaghri et al., 2013). Findings in older children are equally mixed with some showing associations with these variables (Rippe et al., 2016; Ursache et al., 2017) but others finding no association between individual adversity factors and hair cortisol (Gerber et al., 2017; Groeneveld et al., 2013; Karlén et al., 2015; White et al., 2017). Reasons for these differing findings are not clear, however two studies using composite scores of socioeconomic status in Sweden (Karlén et al., 2015) and the Netherlands (Vliegenthart et al., 2016) similar to our methodsfind similar results, suggesting that these composite scores may more clearly identify the cumulative nature of risk to children with attendant cortisol rise compared with examining individual risk factors separately. We think this is crucial to understanding our results.

The finding that maternal-stress factors were less critical to cortisol than other factors in our analysis should give pause for thought, as these are often some of key issues considered when attempting to address problems in early childhood. Similar to our findings, Liu et al examined children at 9 months & 1 year and found no difference in hair cortisol by maternal stress, affect or mood (albeit in a small sample of 41 children) (Liu et al., 2016). However, Palmer et al found that maternal depression & parenting stress were associated with hair cortisol in 1 year old infants in the USA, with some differences in subgroup analyses between racial groups (Palmer et al., 2013).

That saliva cortisol was not associated with childhood adversity is noteworthy, and contrary to our hypothesis. We found very flat slopes in the majority of the sample and it is possible that this contributes to this finding. For example, St John et al describe slopes which are 10 times greater than the decline we describe in under one year olds (Flom et al., 2017; St. John et al., 2017). Having said that, authors do not always report descriptive data, saliva cortisol values are known to vary depending on assay used (Miller et al., 2013) and slopes are generally shallow overall in young children (for example (Watamura et al., 2003)). Another consideration is that whilst the diurnal rhythm is likely to be in place at this age, the HPA axis continues to mature through the first few years of life (Davis and Granger, 2009; Gunnar and Donzella, 2002; Hill-Soderlund et al., 2015). A recent meta-analysis did not find a relationship between adverse events in childhood and saliva cortisol in adulthood (Fogelman and Canli, 2018). Finally, that the findings are discordant between hair and saliva is in line with previous work (Flom et al., 2017) and theoretical understanding that they are assessing different components of the stress response system described earlier (Russell et al., 2012).

Strengths of our study include the measurement of adversities at the time they are occurring, in contrast with the common approach to Adverse Childhood Experiences focussing on adult recall of childhood events (Felitti et al., 1998). We were also able to include a wider range of adversities, some of which are more easily measured when children are young. Our use of modelling tools allowing for analysis of unbalanced numbers of saliva samples per child (Hruschka et al., 2005).

Limitations are that biological measures were restricted to cortisol whilst biological embedding of adversity is likely to occur through multiple factors acting simultaneously. Assessing multiple biological markers simultaneously (for example, “epigenetic clocks”) can provide more detailed estimation of the biological toll of adversity (Belsky et al., 2018; Jylhävä et al., 2017). A specific limitation related to the saliva cortisol is that the first sample of the day was taken at 8am rather than the closer to waking-time, similarly our final sample was taken at 4 pm rather than immediately before bedtime. Similar schedules have, however, yielded expected results in other studies (Adam and Kumari, 2009), so it is far from clear that this is the reason for our null findings.

That hair cortisol was seen to rise with each increase in a wide-range of adversities suggests that efforts to improve this will require a multisectoral approach to both reduce adversities and to design interventions that can protect children who face these – the care for child development curriculum being promoted by WHO/UNICEF is one such example of an intervention promoting stimulation and nurturing care within the household.

In attempting to understand the effects of adversity in early childhood on a broad range of outcomes, a key question relates to ways in which these become biologically embedded, causing suboptimal lifelong health & wellbeing. Simultaneously, programmers implementing early-life interventions require tools that can provide insight into the biological impact of these. Hair cortisol has the potential to be of use in both regards.

In summary, we present for the first time the finding that early life adversity is related to chronic childhood exposure to cortisol in a low/middle-income country. Most children live in LMICs and these countries are where the burden of adversity and suboptimal health & wellbeing is greatest. Action is ongoing worldwide to tackle childhood adversity at societal, community & household levels and our work reiterates its importance in very early childhood, in a low/middle-income country. Further work is needed to develop deeper understanding into ways in which adversity becomes biologically embedded to help refine this action.

## Author contributions

Sunil Bhopal: Conceptualisation, methodology, software, formal analysis, investigation, writing – original draft, writing – reviewing & editing, project administration, funding acquisition. Deepali Verma: Methodology, formal analysis, investigation, writing – reviewing & editing, project administration. Reetabrata Roy: methodology, formal analysis, investigation, writing – reviewing & editing, project administration. Seyi Soremekun – formal analysis, writing – reviewing & editing. Divya Kumar – investigation, writing – reviewing & editing, project administration. Matt Bristow – Methodology, supervision, writing – review & editing. Aparna Bhanushali – validation, investigation, writing – review & editing. Gauri Divan - methodology, investigation, writing – reviewing & editing, supervision, project administration. Betty Kirkwood – Conceptualisation, methodology, formal analysis, investigation, writing – reviewing & editing, supervision, project administration, funding acquisition.

## Conflict of interest

None
