# Supplementary material for: The contribution of childhood adversity to cortisol measures of early life stress amongst infants in rural India: Findings from the early life stress sub-study of the SPRING cluster randomised controlled trial (SPRING-ELS)
Source: Psychoneuroendocrinology. 2019 Sep;107:241–50. doi: 10.1016/j.psyneuen.2019.05.012 (PMC6642338; doi:10.1016/j.psyneuen.2019.05.012)
Supplement: Supplementary file 1 [file mmc1.docx]

**The contribution of childhood adversity to cortisol measures of early life stress amongst infants in rural India: findings from the early life stress sub-study of the SPRING cluster randomised controlled trial**

**(SPRING-ELS)**

**Supplementary File 1: Details of Principal Components Analysis**

| **Adversity type** | **Factor loading** |
| --- | --- |
| Socioeconomic status: lowest quintile (*E) ^a^ | 0.3405 |
| Violence against mother by husband | 0.3090 |
| Mistreatment of mother by other family member or acquaintance | 0.3032 |
| Mother education: none or 1-5 grades (*E) | 0.2986 |
| Older children who live in house hit/punched/kicked/bit child on purpose to make them unhappy | 0.2575 |
| PHQ9 score >=5 or problems described make it very/extremely difficult to do daily activities | 0.2387 |
| Any of mother, father, mother or mother-in-law were “unhappy” when found out child was a girl ^f^ | 0.2372 |
| Mother married under legal age (18 years) | 0.2324 |
| Father education: none or 1-5 grades | 0.2222 |
| Older children who live in house: say anything to make child cry or unhappy (in last week) | 0.2218 |
| HOME score: lowest quintile | 0.2204 |
| Father alcohol use | 0.2081 |
| Family debt | 0.2053 |
| MORS concern: moderate or high | 0.1949 |
| Father occupation: at home, seasonably employed or casual labourer | 0.1886 |
| PHQ problems make it very/extremely difficult to do daily activities | 0.1536 |
| Duke scale: support <=40 | 0.1284 |
| Duke scale: stress >27 | 0.1054 |
| Mother reports death of husband, parent, sibling, child or friend since pregnancy | 0.0714 |
| Food insecurity | 0.0642 |
| Child left with child under 10 years for more than one hour in the past week | 0.0537 |
| Mother seriously injured or ill since pregnancy | 0.0426 |
| Mother-reported child born early | 0.0314 |
| Observed feeding style: very low quality | 0.0148 |
| Child left alone for more than one hour in the past week | 0.0147 |
| Mother & child separated for one week or more | -0.0225 |
| Child admitted to hospital any time after birth | -0.0263 |

Table 1: Factor loading for each of the adversities including in principal components analysis

Figure 1: Scree plot of eigenvalues for principal components analysis performed
